# Assessing a Digital Platform (KOKU-Nut) to Improve Nutrition in Older Adults: Mixed Methods Feasibility Randomized Controlled Trial

**DOI:** 10.2196/87351

**Published:** 2026-08-28

**Authors:** Chloe French, Sorrel Burden, Jaheeda Gangannagaripalli, Emma Stanmore

**Affiliations:** 1 School of Health Sciences, University of Manchester, Jean McFarlane Building, University of Manchester, Manchester, M13 9PY, United Kingdom, 44 01613067856; 2 Northern Care Alliance NHS Trust, Salford Care Organisation, Salford, United Kingdom; 3 NIHR Applied Research Collaboration Greater Manchester, Manchester, England, United Kingdom; 4 Manchester Academic Health Science Centre, Manchester, United Kingdom

**Keywords:** feasibility, digital health, malnutrition, older adults, nutrition

## Abstract

**Background:**

Keep-on-Keep-up Nutrition (KOKU-Nut) is a free, tablet-based digital platform that focuses on increasing physical activity and improving the dietary intake of older adults.

**Objective:**

The aim of this study was to assess the feasibility of using KOKU-Nut among community-dwelling older adults. Feasibility was assessed by considering recruitment and retention rates, acceptability of the intervention, and study design.

**Methods:**

Participants (community-dwelling adults aged ≥65 years) were randomized 1:1 to either the intervention or control group. The intervention group was asked to engage with KOKU-Nut 3 times a week for 12 weeks. Participants in the control group received a leaflet promoting a healthy lifestyle. All participants completed questionnaires at baseline and 12 weeks. A sample of participants was asked to complete an optional interview. The study collected data on dietary intake (24-hour food diary), physical function (grip strength and 5-time sit-to-stand exercise), usability of the intervention (System Usability Scale), and safety (adverse events).

**Results:**

Of 51 participants assessed for eligibility, 31 were randomized, and 28 completed the 12-week follow-up. Of the 14 participants in the intervention group, 10 (71.4%) reported engagement with KOKU-Nut 3 or more times a week. There was no difference in physical function or dietary intake at 12 weeks between participants in the intervention and control groups after adjusting for age and baseline values.

**Conclusions:**

This feasibility randomized controlled trial indicates that the study design was appropriate and acceptable to older adults, as demonstrated by the recruitment and retention rates. This is promising and demonstrates the potential for conducting a future powered randomized controlled trial to assess the effectiveness of KOKU-Nut.

## Introduction

Data from the Office for National Statistics show that, in 2016, there were 11.8 million UK residents aged 65 years and over, representing 18% of the UK population [1]. Projections for 2066 estimate that this will increase by 8% to 20.4 million people [1]. Despite increases in life expectancy, healthy lifespan is not increasing at the same rate, and these additional years are often spent in poor physical and mental health [2].

Malnutrition is common among older adults and often arises from an interplay of physiological, psychological, and social factors, including dysphagia, chewing difficulties, bereavement, social isolation, and mobility limitations, all of which can influence nutritional intake [3-6]. Estimates suggest that approximately 8.5% of community-dwelling older adults are at risk of or experience malnutrition [7]. Observational and experimental evidence demonstrates that a healthy diet can slow disease progression, reduce the rate of functional decline, and prevent undernutrition and frailty [5,8-11], whereas the presence of malnutrition leads to adverse health outcomes, premature mortality, and increased costs on health and social care [12,13]. Older adults living in the community are likely to have greater independence and choice regarding their dietary intake than individuals living in care homes or hospital settings, where meals and food provision are more structured and institutionally regulated [14]. A James Lind Alliance Priority Setting Partnership identified that early intervention in vulnerable groups was the number one research and innovation priority for adult malnutrition [15]. This highlights the need and rationale to support the nutritional intake of older adults to help prevent malnutrition

An aging population with high health care demands [1] coupled with technological advances creates an opportunity for innovative digital solutions to support health care [2,16,17]. Keep-on-Keep-up is a National Health Service–approved digital fall prevention program designed with and for older adults [18]. The platform includes progressive strength and balance exercises [19-21], gamification to improve awareness of bone health and safety in the home, and behavior change techniques to support users in building habits and tracking behaviors. Keep-on-Keep-up Nutrition (KOKU-Nut) is the latest iteration [6,22], providing nutrition advice to complement the strength and balance exercises. Multi-domain interventions appear to have greater benefits for functional outcomes in older adults [23] given that adverse health in older age often encompasses multiple deficits across multiple domains [24].

The aim of this study was to assess the feasibility of conducting a randomized controlled trial using the digital platform KOKU-Nut to improve dietary intake in community-dwelling older adults. Feasibility was assessed by considering recruitment and retention rates and acceptability of the study design. This study also considered the usability, preliminary efficacy, and safety of KOKU-Nut.

## Methods

### Overview

A sequential mixed methods study design was chosen that used quantitative and then qualitative research techniques given the difficulty of evaluating a complex intervention and to maximize the breadth and depth of the research findings. The protocol was previously published [22], and the trial was registered prior to recruitment (NCT05943366). This study was reported in accordance with the COREQ (Consolidated Criteria for Reporting Qualitative Research) guidelines [25] to ensure comprehensive and transparent reporting of the qualitative methods and findings (Multimedia Appendix 1).

### Ethical Considerations

Ethics approval was obtained from the University of Manchester Research Ethics Committee on August 18, 2023 (reference 2023-17372-30569), and this study complied with the Declaration of Helsinki. Written informed consent was obtained from all participants prior to commencement of the research.

### Eligibility Criteria

Participants were included in the study if they were aged 65 years and older, living independently in the community in Greater Manchester, and willing to use an iPad or tablet (their own or one provided by the research team) for the duration of the study. Participants were excluded if they were unable to communicate in English or had a known cognitive impairment.

### Recruitment and Sampling

Men and women aged 65 years and older living independently in the community were recruited on a rolling basis through purposive sampling via local Age UK groups, assisted living facilities, and university networks. Initially, a phone call was arranged to determine eligibility. The researcher verbally explained the aims of the study, how data were collected, and what was required from participants and encouraged participants to ask any questions or raise any concerns.

Recruitment to the qualitative study was done purposefully toward the end of the intervention period to recruit a demographically representative group of participants, including variation in sex, age, and treatment group. Researchers continued to invite participants until data saturation was reached, defined a priori as the point at which no new themes emerged from successive interviews during data coding and charting.

### Randomization

Upon meeting the eligibility criteria and providing written informed consent, participants were randomized into either the intervention or control group by an independent member of the research team using sealedenvelope [26]. Participants were informed of their group allocation after baseline data had been collected.

### Study Intervention

KOKU-Nut [6] is a digital platform that can be downloaded on tablet or iPad devices. The platform includes progressive and personalized strength and balance exercises alongside a suite of educational games, including a nutrition matching pair card game to educate and nudge users to support their dietary intake (see Multimedia Appendix 1 for screenshots). In particular, the educational content focuses on (1) increasing intake of high-quality protein items such as milk, yogurt, and fish; (2) increasing fluid intake to 6 to 8 glasses a day; and (3) educating users on the signs and risks of malnutrition.

Participants in the intervention group were asked to engage with KOKU-Nut in their own time for approximately 30 minutes 3 times a week for 12 weeks. At the baseline visit, participants were supported with downloading the digital platform onto their iPad or tablet. In cases in which participants did not have the necessary device to use the intervention, an iPad (Apple Inc) with KOKU-Nut installed was provided to the participants for the duration of the intervention. The researcher explained and demonstrated the features of the digital platform and assisted with any technical queries. A crib sheet and contact details for the researcher were provided to participants for additional support and to help with technical issues. A family member or friend was invited to attend the baseline visit if the participant wanted additional support. Participants were advised to continue their usual care and treatment.

### Control Group

Participants assigned to the control group received a leaflet developed by Age UK that contained information about the importance of a healthy lifestyle, including the benefits of staying active and eating healthy [27]. Participants in the control group were also advised to continue with usual treatments and care from their health care professionals.

### Data Collection

Written informed consent was collected from all participants prior to collecting the baseline case report file or conducting any assessments at the participants’ homes. The baseline case report file collected data on the participants’ sociodemographic characteristics, as well as the following measures. Health-related quality of life was assessed using the EQ-5D-5L, which considers mobility, self-care, usual activities, pain and discomfort, and anxiety and depression [28]. Each domain is rated across 5 levels of severity, which are combined and converted into a single summary value [29]. The instrument also includes a visual analog scale, where participants rate their overall health from 0 (worst health imaginable) to 100 (best health imaginable). Risk of malnutrition was assessed using the validated Malnutrition Universal Screening Tool [30], and overall risk was assessed as a binary measure to compare those with low vs medium or high risk of malnutrition. Mood was assessed using the short version of the Geriatric Depression Scale [31]. Items were scored dichotomously and summed; overall risk was then assessed as a binary variable so that participants scoring 2 or higher were at risk of depression. These assessments were chosen due to their ease of administration, relevance to the research question, and widespread use across community and clinical settings in the United Kingdom.

During the baseline and 12-week assessments, the researcher also measured grip strength (in kilograms), the time taken to complete 5 sit-to-stand exercises (seconds), and frailty status based on the Clinical Frailty Scale. Further details on these procedures are provided in the study protocol [22].

Participants were asked to complete a 24-hour dietary recall using the online questionnaire Intake24 (version 2) at baseline and 12 weeks [32,33]. Participants were encouraged to follow usual food and drink consumption and then report all food and drink consumed in the online software as accurately as possible. If participants were unable to access Intake24 or experienced technical difficulties, the researcher input this information based on a paper-based version of the participants’ food diary.

The researcher arranged the follow-up assessment 12 weeks after the baseline visits. Baseline measurements were repeated, and participants in the intervention group also reported outcome data related to the usability and acceptability of the intervention, assessed using the technology acceptance model (TAM) [34] and System Usability Scale (SUS) [35].

A subsample of participants completed one-to-one semistructured interviews at the end of the intervention period with a female researcher (CF) experienced in qualitative methodology. Interviews were conducted online or face-to-face based on the preference of the participant, audio recorded, and expected to last no more than 60 minutes. Prior to completing the interview, participants provided additional consent regarding the recording, storage, and use of their interview data. A topic guide was informed by the research aims and similar studies and was used to explore barriers to recruitment, the appropriateness of the outcome measures, and the feasibility of implementing the KOKU-Nut intervention.

### Outcomes

The main outcome was the feasibility, acceptability, and usability of the KOKU-Nut digital platform (Multimedia Appendix 1). Additional outcomes were the potential benefits of engaging with KOKU-Nut, specifically considering protein intake, risk of malnutrition, physical function, and health-related quality of life, as previously described [22].

### Statistical Analysis

Feasibility outcomes and baseline characteristics of the participants were summarized using descriptive statistics, including proportions or means with SDs. Between-group differences in health-related measures at 12 weeks were explored using analysis of covariance adjusting for baseline values of the outcome and participants’ age at recruitment. These analyses were conducted on an exploratory basis to provide preliminary estimates of potential intervention effects. Given the feasibility nature of the study, the sample size was not powered to detect statistically significant differences.

Interviews were transcribed verbatim and then checked by a researcher to ensure accuracy. Transcripts were then managed using NVivo (version 12; Lumivero) [36] and analyzed inductively using the 5 stages of framework analysis [37,38]. The initial stage involved repeatedly reading the transcripts, followed by the development of codes and the identification of an initial thematic framework (CF). Subsequently, data were systematically indexed according to this framework. The fourth stage involved organizing and summarizing the data into thematic charts to facilitate comparison and synthesis. Finally, patterns within the data were interpreted and discussed with all coauthors to generate overarching insights. To enhance analytical rigor, all stages of the process were reviewed and discussed by a minimum of 2 researchers.

## Results

### Sociodemographic Characteristics of the Participants

Table 1 shows the baseline characteristics of all 31 participants enrolled in the study. The mean age was 81.3 (SD 8.3) years, and most participants were female (n=24, 77.4%) and White (n=27, 87.1%). Almost all (n=28, 90.3%) participants were retired, and just under half (n=14, 45.2%) were widowed. Overall, most participants were at a low risk of depression based on the 5-item Geriatric Depression Scale. In total, 12.5% (2/16) of the participants in the intervention group were at risk of depression at baseline, which may have affected motivation to use and engage with KOKU-Nut.

More than half (16/31, 51.6%) of participants were taking 5 or more medications, and there were more participants with polypharmacy in the intervention group (10/16, 62.5%) compared to the control group (6/15, 40%). More than half (16/31, 51.6%) of all participants self-reported one or more falls in the previous 12 months, and fall history was similar between the 2 groups. At baseline, 16.1% (5/31) of the participants were at risk of malnutrition based on a score of 1 or more on the Malnutrition Universal Screening Tool, and there was no difference in risk between the groups. The median frailty score was 4 (IQR 3-5), indicating that the sample was living with very mild frailty. This has been described as “while not dependent on others for daily help, often symptoms limit activities.” A common concern is being “slowed up” and/or being “tired during the day” [39].

**Table 1.** Baseline characteristics of the participants (N=31).

	Intervention (n=16)	Control (n=15)	Total
Sex, n (%)			
Male	4 (25)	3 (20)	7 (22.6)
Female	12 (75)	12 (80)	24 (77.4)
Age (y), mean (SD)	79.9 (8.3)	82.7 (8.4)	81.3 (8.3)
Race, n (%)			
White	13 (81.3)	14 (93.3)	27 (87.1)
Other	3 (18.8)	1 (6.7)	4 (12.9)
Marital status, n (%)			
Married	5 (31.3)	5 (33.3)	10 (32.3)
Widowed	7 (43.8)	7 (46.7)	14 (45.2)
Separated or divorced	4 (25)	3 (20)	7 (22.6)
IMD^a^ (decile 1-10), median (IQR)	5.5 (4-8.75)	6 (4-7)	6 (4-8)
Number of medications, n (%)			
<4	6 (37.5)	9 (60)	15 (48.4)
≥5	10 (62.5)	6 (40)	16 (51.6)
Fall history, n (%)			
Never	8 (50)	7 (46.7)	15 (48.4)
Once	2 (12.5)	3 (20)	5 (16.1)
Twice or more	6 (37.5)	5 (33.3)	11 (35.5)
Requires support with shopping, n (%)			
Independent	9 (56.3)	11 (73.3)	20 (64.5)
Receives some support	5 (31.3)	4 (26.7)	9 (29.0)
Completely dependent	2 (12.5)	0 (0)	2 (6.5)
Internet use, n (%)			
Daily	9 (56.3)	9 (60)	18 (58.1)
Weekly	4 (25)	3 (20)	7 (22.6)
Never	3 (18.8)	3 (20)	6 (19.4)
Technology use, n (%)			
Owns tablet or iPad	5 (31.3)	4 (26.7)	9 (29.0)
Owns smartphone	14 (87.5)	6 (40)	20 (64.5)
Owns laptop or computer	10 (62.5)	10 (66.7)	20 (64.5)
Depression, n (%)			
Low risk (GDS^b^<2)	14 (87.5)	12 (80)	26 (83.9)
At risk (GDS≥2)	2 (12.5)	3 (20)	5 (16.1)
BMI (kg/m^2^), n (%)			
≤24.9	7 (43.8)	8 (53.3)	15 (48.4)
≥25	9 (56.3)	7 (46.7)	16 (51.6)
Risk of malnutrition^c^, n (%)			
Low	13 (81.3)	13 (86.7)	26 (83.9)
Medium-high	3 (18.8)	2 (13.3)	5 (16.1)
Frailty score^d^, median (IQR)	3.5 (2.3-5)	4 (3-5)	4 (3-5)

a IMD: Index of Multiple Deprivation.

b GDS: Geriatric Depression Scale.

c Assessed using the Malnutrition Universal Screening Tool (MUST; low risk=MUST score of 0; medium-high risk=MUST score of ≥1).

d Clinical Frailty Score assessed from 1, representing very fit, to 9, representing terminally ill.

### Feasibility Outcomes: Recruitment and Retention

#### Overview

Between March 2024 and August 2024, a total of 51 potential participants were screened to participate in the study, of whom 8 (15.7%) were excluded because they did not meet the eligibility criteria (n=5 were aged <65 years, n=1 lived outside of Greater Manchester, and n=2 had mild cognitive impairment); a further 12 (23.5%) older adults declined to participate.

**Figure 1.**
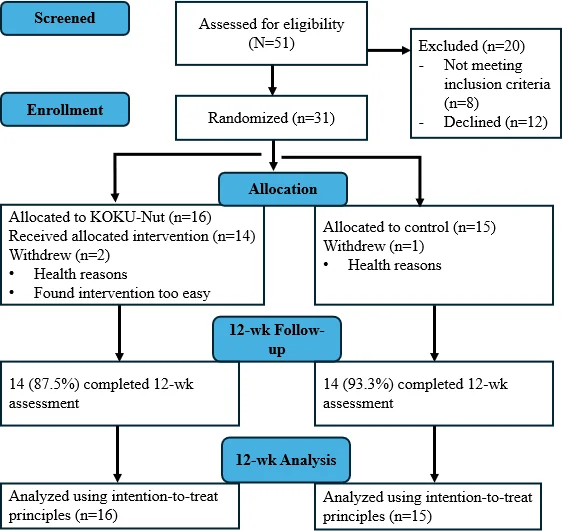
CONSORT (Consolidated Standards of Reporting Trials) flow diagram. KOKU-Nut: Keep-on-Keep-up Nutrition.

In total, 31 older adults were enrolled in the study and randomly assigned to the intervention (n=16, 51.6%) or control (n=15, 48.4%) group (Figure 1). Prior to the 12-week follow-up, 12.5% (2/16) of the participants in the intervention group withdrew (one had a knee operation, and the other found the KOKU-Nut program too easy). In total, 6.7% (1/15) of the participants in the control group withdrew because they had a fall and no longer wanted to take part. As a result, the overall retention rate at the 3-month follow-up was 90.3% (28/31). The study was completed with high retention rates and low levels of missing data; these indicators suggest that the study is feasible (Multimedia Appendix 1). It may be beneficial to have more targeted recruitment strategies to increase recruitment rates and support the conversion of eligible to enrolled participants in a future trial.

Ten participants completed an interview, 6 of whom were in the intervention group, and the remaining 4 were in the control group. Interviews were conducted face-to-face at the participants’ homes and lasted for a mean of 31.5 (SD 12.1; range 18-51) minutes. Framework analysis of the dataset uncovered 3 key themes.

#### Theme 1: Acceptability of the Study

Participants found the questionnaires and physical function tests acceptable; however, there was some resistance regarding the 24-hour dietary recall. The experience of using the online software was variable: some found it interesting to consider the different portion sizes, whereas others were unable or did not want to use the online software and so completed an interviewer-led version. Participants also reported changing their intake to facilitate the recording process or having difficulty entering certain foods:

I was very interested in it [Intake24 survey] and the way it was done, particularly with the measurement of quantities and the options you were given which I thought were very helpful...and I actually enjoyed it.

[Female participant; control group]

I had a word with a couple of people and described what I had done so far, they found the idea of the meal report off-putting.

[Female participant; intervention group]

I omitted some foods as it was tedious to complete, especially when making food from scratch. I didn’t know how to enter homemade soup or cake.... It felt like it was tailored towards someone who would buy a lot of pre-packaged foods, also it doesn’t ask about salt and pepper.

[Female participant; intervention group]

First time I picked the simplest meal that I ever eat.... But the second time I picked a slightly more complicated one.

[Female participant; control group]

In general, the intervention (KOKU-Nut) was acceptable, beneficial, and enjoyable, although there were still some concerns about the digital aspect:

Have found that since making changes I have more energy. Used to experience an afternoon slump around 3pm but now I feel more energised.

[Female participant; intervention group]

For people that are familiar with tablets it would be fantastic.... But I’ve got friends in their seventies and eighties who don’t use smartphones!

[Male participant; intervention group]

#### Theme 2: Healthy Behaviors

Many participants in both the intervention and control groups highlighted that taking part in the study made them reflect on their health and their current dietary choices, which made them want to make positive changes. Participants in the intervention group found that using KOKU-Nut prompted them to consider their health and start a new routine:

Since [starting the study, I] made small changes to diet and exercise routine and app nudged me in the right direction.

[Male participant; intervention group]

I want to be active and independent for as long as possible and this provided encouragement to make small changes.

[Female participant; control group]

I now try to get protein at each meal.... So yes, I’ve introduced yoghurt on top of the porridge.

[Male participant; intervention group]

Since starting the study have made an effort to increase protein so now, I check the packaging to look for grams of protein to ensure that having protein with all meals.

[Female participant; intervention group]

KOKU has made me more conscious of cooking and what we can and should be having for a healthy diet.

[Female participant; intervention group]

Participants also recognized the importance of habit formation and that incorporating KOKU-Nut into their routine helped with engagement:

I think it might be good for people to think about where and when they use it and how it can fit in with their routine.

[Female participant; intervention group]

Participants in the control group also highlighted that the study prompted them to make more positive lifestyle changes, with one participant saying the following:

And then I was thinking it’s not just your physical health, you’ve got to think of your mental health as well. So, I’ve signed up to do Tai Chi on a Monday.

[Female participant; control group]

However, participants identified the limitations of using a booklet to support behavior change:

The thing with a booklet is, you read it and then you put it to one side and then that’s it. And if you need prompting there isn’t anything there.

[Female participant; control group]

#### Theme 3: Future Considerations

Participants highlighted additional features that they thought would add value to KOKU-Nut and support them in making positive changes to their diet. This included a myth-busting information section, an example week’s menu, or recipe ideas with video and written content:

Due to my hypertension I never know if I can have cheese or yoghurt so it would be great to have information about if I can eat these foods.

[Female participant; intervention group]

It would be great to have more nutrition advice and content for example to explain what a high protein diet actually is.

[Male participant; intervention group]

I’d find it very useful to have videos or written recipes with an example weeks menu...or even just some recipe inspiration.

[Female participant; intervention group]

### Additional Outcomes

#### Usability and Safety of the Intervention

Of 14 participants in the intervention group, 10 (71.4%) self-reported engagement with KOKU-Nut 3 or more times a week as suggested at the start of the intervention. Of 16 participants in the intervention group, 11 (68.8%) received an iPad provided by the research team, and 5 participants (31.2%) had their own devices. Based on the SUS, KOKU-Nut had very good usability, with a mean score of 79.7 (SD 15.2), above the industry standard average of 68 [40]. Participants with their own iPads provided slightly higher and more consistent usability scores (mean 82, SD 6.2) compared to participants who did not have their own iPad (mean 78.6, SD 18.7). There were no adverse events reported over the 12-week period.

#### User Experience

Fourteen participants considered the acceptability of KOKU-Nut based on the TAM as this was not completed by the 2 participants who withdrew from the study. Participants rated the nutrition game highly on perceived ease of use, perceived usefulness, attitude toward use, and intention to use (Multimedia Appendix 1). There was moderate correlation between perceived usefulness and attitude toward and intention to use (Table 2). This is important as the TAM suggests that there is an association between the user’s intention to use the technology and their actual use behavior [41].

**Table 2.** Technology acceptance model questionnaire for community-dwelling older adults and their experience of using Keep-on-Keep-up Nutrition (KOKU-Nut). Each aspect was scored on a scale ranging from 0 to 7 such that 7 represented “most favorable” (ie, highest level of perceived usefulness). There were missing data from 2 participants in each case due to withdrawals.

	Perceived ease of use	Perceived usefulness	Attitude toward use	Intention to use
Scores, median (IQR)	6.3 (5.4-7.0)	5.3 (4.9-5.9)	6.9 (5.9-7.0)	5.3 (3.7-6.3)
Correlation with intention to use, Pearson r (*P* value)	0.3 (0.27)	0.5 (0.05)	0.65 (0.01)	—^a^

a Correlation between intention to use and intention to use is not reported.

### Preliminary Effectiveness of the Intervention

Participants undertook assessments of functional ability, dietary intake, and quality of life at baseline and the 12-week follow-up. An exploratory intention-to-treat analysis was conducted, but as this was a feasibility study, it was not sufficiently powered to detect significant differences between groups.

There was no difference in grip strength or time taken to complete 5 sit-to-stand exercises at the follow-up assessment between groups after adjusting for age and baseline values.

EQ-5D-5L scores were similar between the groups at baseline and reflect the norms for this age group [42]. After the 12-week intervention, there was a difference in EQ-5D-5L scores using analysis of covariance (*F*_1,27_=7.26; *P*=.01), again adjusting for age and baseline values. Participants in the intervention group had higher mean EQ-5D-5L scores than participants in the control group (at 12 weeks; 0.72, 95% CI 0.67-0.78 vs 0.62, 95% CI 0.57-0.78 in the control group; *P*=.01).

Table 3 shows that baseline mean protein intake was above the reference nutrient intake of 0.75 grams per kilogram of body weight per day [43,44]. There was no difference in energy (kilocalories per day) or protein (grams per kilogram of body weight per day) intake at the follow-up assessment between participants in the intervention and control groups when adjusting for baseline values (Table 3).

**Table 3.** Physical function and nutrition outcomes at baseline and 12 weeks by treatment group in community-dwelling older adults (N=31).

Parameter	Baseline, mean (SD)	Week 12, mean (SD)	*P* value
Grip strength (kg)			.77
Intervention	20.19 (11.95)	20.31 (10.07)	
Control	18.33 (6.34)	18.47 (6.39)	
5-STS^a^ (s)			.27
Intervention	26.08 (16.98)	24.27 (17.10)	
Control	29.82 (17.07)	30.53 (16.46)	
Energy intake (kcal per d)			.80
Intervention	1459 (468)	1415 (455)	
Control	1548 (468)	1432 (227)	
Protein intake (grams per kilogram of body weight per d)			.87
Intervention	1.00 (0.29)	1.01 (0.32)	
Control	0.96 (0.33)	0.97 (0.24)	
EQ-5D-5L (−0.59 to 1)			.01
Intervention	0.68 (0.27)	0.74 (0.21)	
Control	0.64 (0.22)	0.60 (0.22)	
VAS^b^ (0-100)			.61
Intervention	81.10 (18.53)	79.94 (13.96)	
Control	77.73 (17.63)	75.33 (15.41)	

a 5-STS: 5-time sit-to-stand exercise.

b VAS: visual analog scale.

## Discussion

### Principal Findings

This 12-week intervention informed by the Medical Research Council guidelines for the development and evaluation of a complex intervention [45] in older adults living in the community in Greater Manchester was feasible and acceptable. Participants reported high usability of KOKU-Nut and found the digital platform acceptable and easy to use, as demonstrated by the SUS [35] and TAM [34]. The current study had retention rates above 90%, and framework analysis of the interviews demonstrated that the study design, assessments used, and allocated treatment groups were acceptable to participants.

This study managed to recruit an at-risk population with low attrition at 12 weeks (3/31, 9.7%). Participants had a mean age of 81.3 (SD 8.3) years, ranging from 70 to 96 years. At baseline, more than half (10/16, 51.6%) of the sample had fallen at least once in the previous 12 months, 16.1% (5/31) were at risk of malnutrition, and most of the participants (median score 4, IQR:3,5) were considered vulnerable according to the Clinical Frailty Scale.

The KOKU-Nut intervention had no impact on energy or protein intake and physical function outcomes at 12 weeks; however, this was a feasibility study with a small sample size and, thus, not powered to detect statistical differences. In addition, interpretation of these findings is further limited by the baseline characteristics of the sample, who were well nourished with adequate protein intake of approximately 1 gram per kilogram of body weight per day at baseline [46]. This introduces a potential ceiling effect whereby participants were already meeting dietary requirements and limits the scope for measurable improvement. Future trials should consider additional eligibility criteria to recruit individuals with suboptimal dietary intake at baseline to enhance sensitivity to change and increase the likelihood of detecting a meaningful intervention effect. Increasing protein intake can support the anabolic effects of strength training and minimize age-related muscle loss, and this has previously been demonstrated in older adults [47], including those with frailty [48] and sarcopenia [49]. However, these dietary interventions did not use a digital platform, and very few apps simultaneously improve dietary intake and support strength training in this population [50]. The study by van den Helder et al [51] demonstrates that combining a home-based exercise program delivered via a tablet-based app with dietary protein counseling delivered via face-to-face dietitian sessions can effectively increase protein intake in older adults. Additional work should focus on the development of a digital tool to support dietary intake among older adults. Additional educational games, high-protein recipes, and goal-setting features are likely to support users in changing their behavior, promote protein intake, and increase the effectiveness of KOKU-Nut.

Most participants in the intervention group (10/14, 71.4%) self-reported using KOKU-Nut 3 or more times a week, demonstrating a satisfactory level of adherence to the intervention. This indicates that older adults can regularly and independently engage with a digital platform such as KOKU-Nut even without prior experience of using an iPad or tablet. A feasibility study with 25 older adults found that 80% of participants regularly used a tablet-based self-monitoring nutrition app (Appetitus) [52]; this complements findings from the current study demonstrating the potential to engage older adults with digital nutrition tools, but further research is required on long-term use and effectiveness. Despite high levels of engagement and usability in the current study, future digital interventions may benefit from increased involvement of caregivers or family members, particularly for those with lower digital literacy, to support confidence, engagement, and uptake.

Unlike the interactive KOKU-Nut intervention, the leaflet-based control condition provided a relatively passive form of intervention delivery. Leaflets are often provided to this population for health guidance and information and, thus, reflect treatment as usual; however, an active control condition may provide greater insight into the effectiveness of the KOKU-Nut intervention. Moreover, qualitative findings indicated perceived behavior change in participants across both trial arms, suggesting that study participation itself may have influenced diet and lifestyle behaviors. This is likely influenced by self-monitoring effects associated with completing assessments and heightened motivation due to involvement in a research study.

This study had many strengths. The approach aligns with the Medical Research Council framework for the development and evaluation of complex interventions [45]. The mixed methods design provides a more comprehensive and nuanced understanding of the feasibility and delivery of the study compared to either methodology alone. Attrition bias was low given that this study had a 90.3% (28/31) retention rate, with only 3 participants dropping out during the 12 weeks. This demonstrates that it was possible to recruit and retain a group of older adults who are at a high risk of falls and often not included in research. Moreover, KOKU-Nut is a minimally invasive digital intervention, so it would be suitable for wide-scale implementation, and a small effect in a large population has the potential to have a big overall effect with low cost or risk.

There are also several limitations that must be acknowledged. Participants were unaware of their allocation until after completing baseline assessments; however, randomization had already been performed prior to the assessment. The lack of allocation concealment may introduce selection bias. This study was not blinded due to resource constraints that, again, may introduce detection bias. This study could have compared the use of KOKU-Nut with that of a generic health app that was not designed for this population to blind the intervention. However, due to limited access to iPads, the control group received a leaflet explaining the benefits of healthy lifestyle habits, and this is likely to better represent standard practice or usual care. The nutrition game was embedded into the Keep-on-Keep-up app, and therefore, it was not possible to specifically look at engagement with the nutrition component or identify whether self-reported use and improvements in quality of life were related to the nutrition game or a different part of the digital platform. Engagement with the digital platform was self-reported, so future studies should aim to develop a dashboard or use Google Analytics data to capture use time objectively. Methodologies to inform the best practice to conduct a gold-standard clinical trial for a digital intervention should be prioritized. Although many assessments were objective, dietary recall is often subject to recall and response bias from underreporting and poor memory. Dietary assessment is prone to misreporting of portion sizes and foods for social approval [53-55]. Moreover, the qualitative work highlighted challenges with completing the 24-hour recall, including burden, difficulties entering complex or homemade foods, and a tendency to simplify intake to facilitate recording. These issues suggest that dietary assessment tools may require greater flexibility and user support to reduce respondent burden and improve data completeness.

### Conclusions

Overall, this feasibility randomized controlled trial demonstrates that the study design was appropriate and acceptable to older adults, as indicated by the recruitment and retention rates as well as the themes that emerged from the interviews. This is promising and demonstrates the potential for conducting a larger study in the future that will be powered to detect effectiveness.

KOKU-Nut had good usability, acceptability, and engagement according to the SUS, TAM, and self-report use, indicating that the intervention was designed appropriately to engage this population. However, the exploratory outcomes exhibited no difference in physical function or dietary intake. This demonstrates that further developments should be made to KOKU-Nut to support dietary change before conducting a larger study to see whether KOKU-Nut can lead to clinical and meaningful impact.

## Supplementary material

10.2196/87351 Multimedia Appendix 1 Screenshots, study assessment schedule, feasibility progression criteria findings, and technology acceptance model results for the Keep-on-Keep-up Nutrition trial

10.2196/87351 Checklist 1 CONSORT checklist.

## Abbreviations

COREQ: Consolidated Criteria for Reporting Qualitative Research
KOKU-Nut: Keep-on-Keep-up Nutrition
SUS: System Usability Scale
TAM: technology acceptance model
