# Supplementary material for: Assessing a Digital Platform (KOKU-Nut) to Improve Nutrition in Older Adults: Mixed Methods Feasibility Randomized Controlled Trial
Source: JMIR Aging. 2026 Aug 28;9:e87351. doi: 10.2196/87351 (PMC13524216; doi:10.2196/87351)
Supplement: Supplementary file 2 [file aging-v9-e87351-s001.docx]

**Supplementary material**

**Supplementary material 1:** Screenshots of Keep-on-Keep-up Nutrition (KOKU-Nut) game

**Supplementary material 2**: Schedule for measurements throughout the study period

| **Measure/ assessment** | **Method of assessment** | **Baseline** | **12-weeks** |
| --- | --- | --- | --- |
| Dietary assessment | 24-hour food diary (INTAKE 24) | X | X |
| Physical function | 5-Sit-to-stand, grip strength | X | X |
| Quality of life | EuroQol | X | X |
| Mood | Geriatric depression scale | X | X |
| Malnutrition assessment | Malnutrition Universal Screening Tool | X | - |
| Frailty assessment | Clinical frailty scale | X | - |
| Participants experience | Interviews | - | X |
| Feasibility | Self-reported use of KOKU-Nut, recruitment/ retention rates | - | X |
| Usability of KOKU-Nut | System usability scale | - | X |
| Acceptability of KOKU-Nut | Technology acceptance model | - | X |
| Adverse events | Self-reported | - | X |

**Supplementary material 3:** Progression criteria for feasibility randomised controlled trial

|  | **Proceed with RCT** | **Do not proceed unless changes are possible** | **Findings** |
| --- | --- | --- | --- |
| **Eligibility of participants** | More than 50% of participants eligible | Less than 20% participants eligible | 84.3% of participants screened eligible |
| **Feasibility of participant recruitment** | More than 50% of eligible participants recruited | Less than 25% of eligible participants recruited | 72.1% of eligible participants enrolled |
| **Feasibility of participant retention** | Less than 20% loss to follow-up | More than 40% loss to follow-up | 9.7% of sample lost to follow-up |
| **Intervention implementation** | More than 70% self-report use of intervention at least 3 times a week | Less than 30% self-report use of intervention at least 3 times a week | 71.4% use KOKU-Nut ≥3 times a week |
| **Intervention acceptability** | Delivery of intervention judged strongly feasible by intervention data | Delivery of intervention judged possibly feasible by qualitative data | Interviews, system usability scale and technology acceptance model show KOKU-Nut was acceptable |
| **Outcome acceptability** | More than 80% outcome measure completion | Less than 70% outcome measure completion | 9.7% of participants unable to complete 5-STS. 9.7% of participants withdrew so no follow-up data |

*5-STS: 5 times sit-to-stand*

**Supplementary material 4**

PEOU- perceived ease of use, PU- perceived usefulness, ITU- intention to use
